# A Functional Study of Human Inflammatory Arthritis Using Photoacoustic Imaging

**DOI:** 10.1038/s41598-017-15147-5

**Published:** 2017-11-03

**Authors:** Janggun Jo, Guan Xu, Meng Cao, April Marquardt, Sheeja Francis, Girish Gandikota, Xueding Wang

**Affiliations:** 1 Department of Biomedical Engineering, University of Michigan, Ann Arbor, Michigan 48109 USA; 2 Department of Radiology, University of Michigan Medical School, Ann Arbor, Michigan 48109 USA; 3 Department of Electronic Science and Engineering, Nanjing University, Nanjing, 210093 China; 4 Division of Rheumatology, Department of Internal Medicine, University of Michigan School of Medicine, Ann Arbor, Michigan 48109 USA

## Abstract

By using our dual-modality system enabling simultaneous real-time ultrasound (US) and photoacoustic (PA) imaging of human peripheral joints, we explored the potential contribution of PA imaging modality to rheumatology clinic. By performing PA imaging at a single laser wavelength, the spatially distributed hemoglobin content reflecting the hyperemia in synovial tissue in metacarpophalangeal (MCP) joints of 16 patients were imaged, and compared to the results from 16 healthy controls. In addition, by performing PA imaging at two laser wavelengths, the spatially distributed hemoglobin oxygenation reflecting the hypoxia in inflammatory joints of 10 patients were imaged, and compared to the results from 10 healthy controls. The statistical analyses of the PA imaging results demonstrated significant differences (p < 0.001) in quantified hemoglobin content and oxygenation between the unequivocally arthritic joints and the normal joints. Increased hyperemia and increased hypoxia, two important physiological biomarkers of synovitis reflecting the increased metabolic demand and the relatively inadequate oxygen delivery in affected synovium, can both be objectively and non-invasively evaluated by PA imaging. The proposed dual-modality system has the potential of providing additional diagnostic information over the traditional US imaging approaches and introducing novel imaging biomarkers for diagnosis and treatment evaluation of inflammatory arthritis.

## Introduction

Longstanding active inflammatory arthritis causes significant disability, leading to substantial activity limitation, work disability, reduced quality of life, and high health-care costs^1^. The development of optimal and personalized treatment options with powerful new drugs to prevent disease progression will depend upon better functional imaging for early diagnosis and assessing the treatment outcome. Physiological states, such as blood flow, blood volume, and blood oxygenation, are closely related to the metabolic state of inflamed joint tissue and hence can be important diagnostic parameters of arthritis. The intense hyperemia of affected joint is not only from the hypervascularization but also the dilatation of veins and capillaries, and is an essential step in the inflammatory cascade. Due to the increased metabolic demand of the inflamed synovium and the relatively inadequate oxygen delivery of the inflamed joint, the inflamed synovium shows profound hypoxia that is another functional feature of synovitis^2^. Recent study discusses a link between localized areas of hypoxia in rheumatoid arthritis synovium and inflammation activation of synovial fibroblasts that is accompanied by increased tissue invasiveness^3,4^. Hypoxia induces neoangiogenesis, thus expanding the extent of synovitis^5^, but the degree of neoangiogenesis is unlikely to fully resolve the hypoxia.

Development and application of new technologies that can identify and evaluate these pathological biomarkers, namely neoangiogenesis, hyperemia, and hypoxia, may shed new light on research and clinical management of inflammatory arthritis. Magnetic resonance imaging (MRI) has been used to identify increased vascularity related to inflammatory arthritis and assess the hemoglobin oxygenation in arthritis with a rabbit model^6,7^. However, the high cost and controlled access can limit the widespread use of MRI for general clinical screening and diagnosis. Ultrasound (US) with Doppler function provides high resolution anatomic imaging and high sensitivity in identifying abnormal blood flow^8,9^, and has shown capability to distinguish between active and inactive synovitis^10,11^. Color Doppler US, however, is more sensitive to the fast blood flow in relatively large vessels. Slow blood flow in smaller capillaries, which are more clinically and pathologically relevant to early active synovitis^12^, could be missed by color Doppler US. Power Doppler, with less dependence on the velocity and direction of flow, has been introduced for improved sensitivity to slow blood flow^13,14^. Nonetheless, both color and power Doppler measurements require extensive training, and are operator dependent and hardly quantifiable. Moreover, US imaging is not capable of evaluating hypoxia as another important biomarker of inflammatory arthritis.

In contrast to assessing the blood flow relative to the transducer array in Doppler US, optical imaging quantifies the presence of blood by assessing the absolute optical energy absorbed by hemoglobin. The concentrations of oxygenated and deoxygenated hemoglobin can also be resolved in multi-spectral optical imaging by utilizing the differences between their unique optical absorption spectra. Pure optical imaging has demonstrated the desired sensitivity in identifying the neovascularity in inflammatory joints of the digits^15,16^. Spectroscopic optical imaging has identified inflammation and the variations of hemoglobin oxygen saturation level in finger arthritis^17,18^. The inflammation has also been visualized in optical imaging by introducing exogenous contrast agents^19^. One problem hindering the application of optical imaging to clinical management of arthritis is the limited spatial resolution as a result of the strong light scattering in biological tissues.

Photoacoustic (PA) imaging (PAI) is an emerging, non-ionizing, non-invasive, low cost technology, with the capability of both structural and functional imaging^20–24^. By acquiring the PA waves with US systems and then conducting image reconstruction based on the back-projection of the acquired signals, the anatomy dependent on optical contrast in biological tissue can be mapped at ultrasonic resolution^25^. PAI has demonstrated the capability of resolving the tissue structures with optical contrasts in deep healthy human finger joints^26–29^, and identifying inflammatory arthritis in animal models^30–32^. Real-time parallel PA and US imaging of a target joint has been achieved by integrating a laser system to a commercially available research US platform equipped with a GPU card^23^. The feasibility of using a hand held probe integrating laser diode and transducer array connected to a portable US system for inexpensive, real-time dual-modality US and PA imaging of human finger joints has been explored^33^. Cross-sectional imaging of human finger has also been achieved by using a customer-built PA and US dual-modality system working with a concave array with large angular coverage^34^. These dual-modality systems could provide naturally co-registered morphological and physiological information, and may assist in comprehensive understanding of the pathophysiology in arthritic finger joints.

The goal of this study, by using the proposed PA and US dual-modality imaging system, is to explore the feasibility in detection of inflamed human metacarpophalangeal (MCP) finger joints by evaluating the increased hemoglobin content (i.e., hyperemia) and the decreased hemoglobin oxygen saturation (i.e., hypoxia) in synovium. Being able to detect the combination of these disease biomarkers that are not identifiable by traditional US could be valuable in early diagnosis and treatment assessment of inflammatory arthritis. B-scan PA and US images as well as Doppler US images in this study were acquired under the guidance of a fellowship trained musculoskeletal radiologist with over 10 years of experience. Doppler US imaging was employed as the gold standard for identifying inflammatory arthritis patients and healthy control subjects. Statistical analyses of the PA results from inflammatory arthritis patients and healthy control subjects were performed to validate the capability of PAI in identifying active synovitis in the human finger joints by assessing hyperemia and hypoxia.

## Materials and Methods

### PA-US dual imaging system

**Figure 1:**
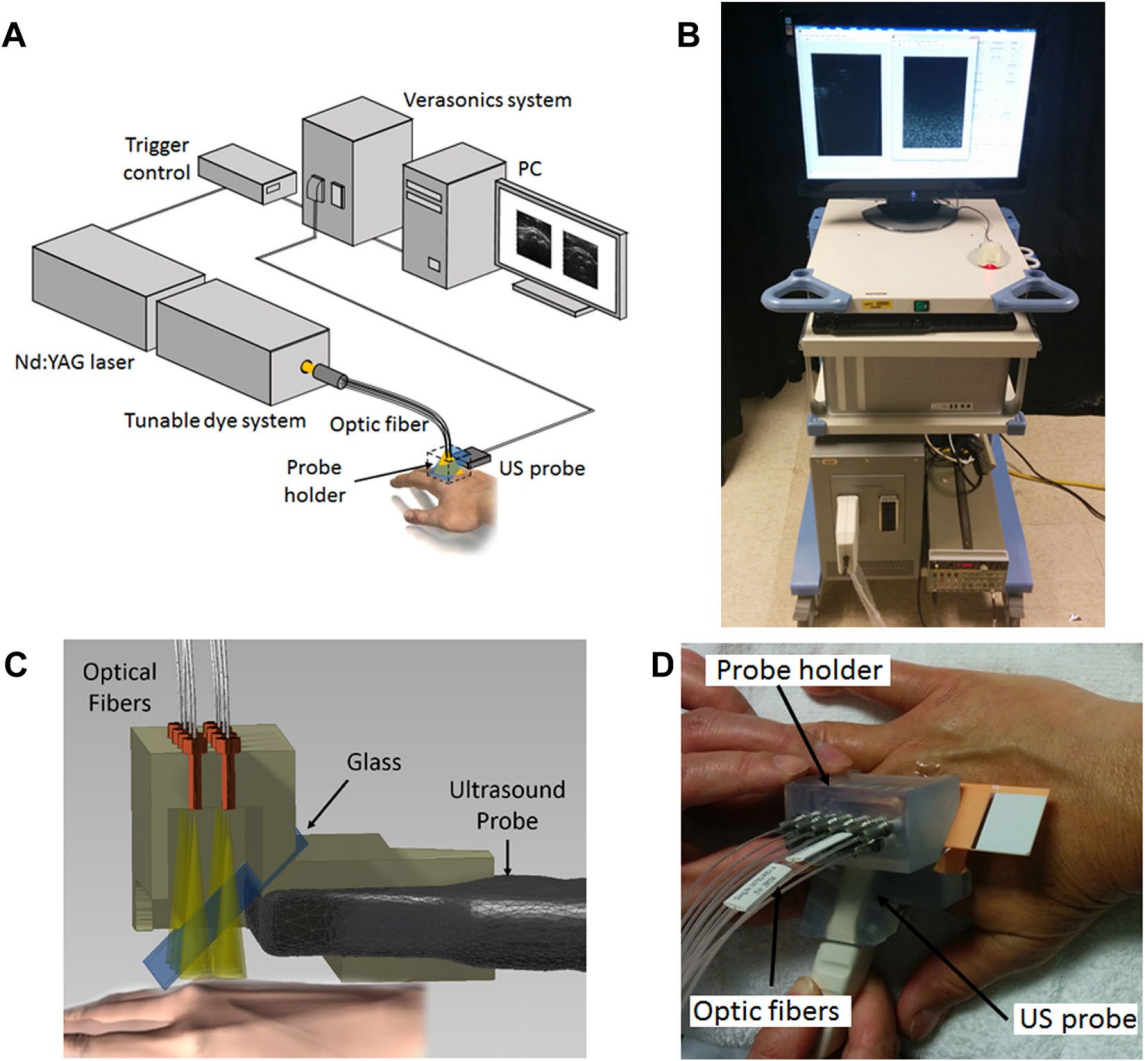
(**A**) Schematic of the PA-US dual system for arthritis imaging. (**B**) Photograph of the system console. (**C**) Schematic showing the designed probe which holds an ultrasound linear transducer array and an optical fiber array. An optical transparent acoustic reflector is used for merging the light beam and ultrasound beam. (**D**) A photograph taken during the scan of a patient MCP joint.

Figure 1A shows the schematics of the PA-US dual-modality joint imaging system built on a commercially available research US platform (V1, Verasonics, Kirkland, WA). The system can acquire 2D B-scan PA and US images simultaneously, both in truly real-time fashion at a frame rate of 10 Hz, as described in a previous publication^23^. PA and US images were acquired using a linear probe (CL15-7, Philips, Andover, MA) working at the default central frequency of 8.9 MHz set by the US platform. A tunable dye laser (ND6000, Continuum, Santa Clara, CA) pumped by the second harmonic of a pulsed neodymium-doped aluminum garnet (Nd:YAG) laser (Powerlite, Continuum, Santa Clara, CA) was used as the primary illumination source for PAI. Another optical parameters oscillator (SLOPO, Continuum, Santa Clara, CA) pumped by an Nd:YAG laser (Surelite, Continuum, Santa Clara, CA) was later integrated for double-wavelength PAI. At the single-wavelength PAI mode, the laser repetition rate was set at 10 Hz. At the double-wavelength PAI mode, the repetition rates of both lasers were set at 5 Hz. The triggering of the secondary laser was delayed by 100 ms after the primary laser, formulating alternating illumination at 10 Hz. Before scanning of each joint, the output pulse energy from each laser was measured continuously over 5 min by a laser power meter (OPHIR, North Logan, UT) to ensure that the laser was stable. For double-wavelength PAI, the average pulse energies from both lasers were recorded and then used later for calibrating the PA images at the two wavelengths before quantifying the hemoglobin oxygenation. Considering that the pulse-to-pulse energy fluctuations of both lasers were relatively small (<5%), no real-time monitoring of pulse energy was conducted. However, for each joint, 10 independent hemoglobin sO_2_ maps resulting from 10 pairs of PA images at the two wavelengths were averaged to reduce the possible error resulting from laser fluctuation.

Based on theoretical analysis and the experiment on a cadaver hand, the optical wavelengths for the specific scenario of PAI of human finger joint were optimized, as the details shown in Supplementary Information (S1). In this study on human peripheral joints, single-wavelength PAI was performed at 580 nm; while double-wavelength PAI was performed at 576 nm and 584 nm. At each of the wavelengths, the light fluence on the skin was approximately 10 mJ/cm^2^. This applied light fluence was well below the 20 mJ/cm^2^ safety limit by the American National Standard Institute (ANSI) at the selected wavelengths.

### Imaging geometry

To accommodate the clinical needs, a handheld probe design based on an optically transparent acoustic reflector was adopted. As shown in Fig. 1C, the laser was coupled into an optical fiber bundle (CeramOptec Industries Inc, East Longmeadow, MA) which consists of 11 fibers each with 1 mm diameter. At the output end, the fibers were distributed evenly along two lines to deliver homogenous light illumination on the skin surface. A 3D printed frame integrated the fiber optics for light delivery and the US probe. To fuse the light beam and the ultrasonic beam, an optically transparent acoustic reflector (a glass slide, 1 mm of thickness, 1.52 of refractive index, 12–550–15, Fisher Scientific) was used. The gaps between the skin surface, the acoustic reflector, and the US probe were all filled with US coupling gel. During the scanning procedure, the light transmitted through the acoustic reflector and illuminated the target joint. Then, the PA signals generated in the joint, after being reflected 90^o^ by the acoustic reflector, were received by the US probe. Similarly, the transmission and receiving beams in the conventional pulse-echo US imaging were also reflected 90^o^.

### Recruitment of patients and healthy control subjects

All procedures in this study were approved by the Institutional Review Board of University of Michigan Medical School, and all methods were performed in accordance with the guidelines and regulations. All healthy and arthritic subjects were provided written informed consent. The patients involved in this study were men and women over 18 years old, with apparent swelling and pain in at least one of their finger joints. Arthritic MCP joints that are most common in arthritis patients were identified by board certified rheumatologists at the University of Michigan Rheumatology Clinic following the American College of Rheumatology (ACR) criteria. Unequivocal cases were intentionally selected as an initial examination of the capability of PAI in identifying the inflammations in finger joints. The active synovitis and enhanced blood flow in the MCP joints were confirmed by Doppler US. To be used as control, healthy subjects with no symptoms and no history of inflammatory arthritis were recruited. The positive Doppler US results of the arthritic joints and the negative Doppler US results of the healthy controls were confirmed by a board certificated radiologist. In the earlier phase of this study involving single-wavelength PAI, the images were acquired from 16 patients and 16 healthy control subjects. In the later phase of this study involving double-wavelength PAI, the images were acquired from another group of subjects including 10 patients and 10 health controls.

### Scanning procedures

During the scanning procedure, the patients are comfortably seated with the scanned hand lied comfortably on a flat surface. During the imaging procedure, laser safety glasses were worn by the patients to avoid potential eye damage. Used as a gold standard for validating that a target joint is either normal or has active inflammation, Doppler US images of the target joint were acquired. Because of our PA-US dual system is not capable of Doppler imaging, the US Doppler images were acquired by a commercial US unit (Z.ONE PRO, ZONARE, Mountain View, CA) working with a linear probe (L14–5W, ZONARE). For Doppler US, the pulse repetition frequency was 1500 Hz and the scale was 10 to −10 cm/s. Following the Doppler US, the target joint was scanned by the PA-US dual system. The imaging plane where prominent blood flow had been found during the Doppler US was revisited. The total scanning time including both Doppler US and PA-US imaging of each joint was less than 10 minutes.

### Co-registration of US and single-wavelength PA images

**Figure 2:**
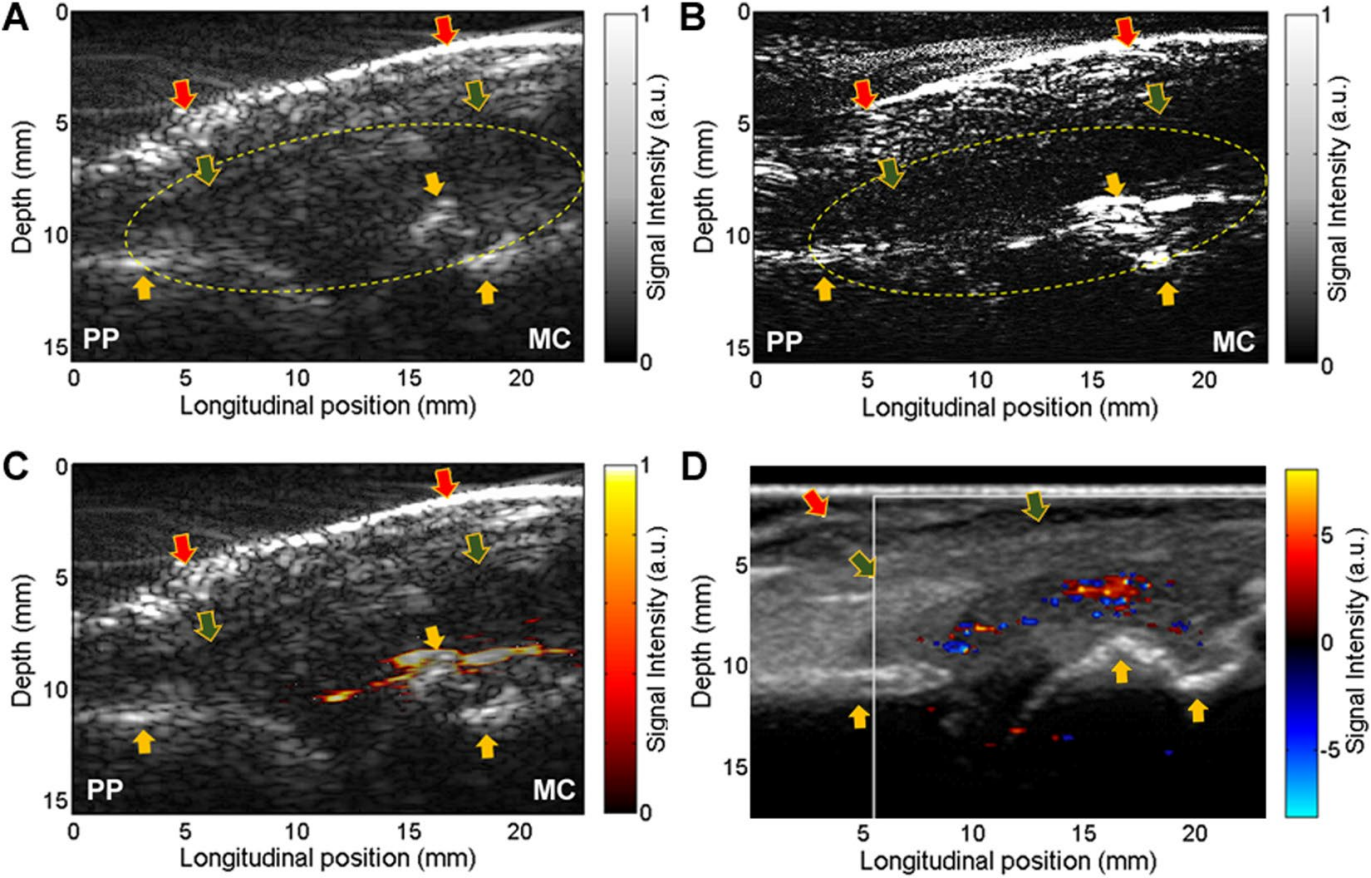
PA and US dual imaging of a right hand 2^nd^ MCP joint affected by inflammatory arthritis. PP: proximal phalanges. MC: metacarpals. (**A**) Gray scale B-mode US image. (**B**) Gray scale PA image of the same imaging plane. (**C**) Pseudo-color PA image superimposed on the gray scale US image, demonstrating the active vascularity in the joint. (**D**) US Doppler image acquired by a commercial US unit confirming the active synovitis in the studied joint. Yellow arrow: bone; Green arrow: tendon; Red arrow: skin.

As an example, Fig. 2 shows a pair of PA and US B-scan images of a human MCP joint with active synovitis. Each PA image was normalized by dividing the intensity of each pixel to the average pixel intensity in the bone area. The normalized color scale makes the PA results from different subjects more comparable. In addition, the intensity map reflected by a PA image before normalization is a product of the spatially distributed light fluence and the spatially distributed tissue optical absorption. The relative contrast among different articular tissues, as presented by a normalized image, is less susceptible to variables including not only laser energy but also patient’s physical parameters such as finger size and skin color. All these parameters can also affect the amplitudes of PA signals from articular tissues.

To better present the vascular information in the PA images, the PA and US images were combined by conducting the following steps: 1) Performed by a board certified musculoskeletal radiologist, a region of interest (ROI) surrounding the target joint was selected in both PA and US images, as shown by the dashed circles; 2) In the normalized PA image, only pixels with intensities over 0.4 (i.e., pixels with intensities higher than 40% of the average intensity in bone area) were kept to remove the system noise and the signals from background tissue absorption. The rationale of using 0.4 as the cutoff threshold to isolate the vasculature in the joint is described in detail in Supplementary Information (S2); 3) The bone structure presented well in US image was extracted and removed from PA image; and 4) The remaining pixels in the PA image were presented in pseudo-color and then superimposed on the gray-scale US image, as an example PA-US combined image shown in Fig. 2C. This post processing procedure took less than 10 minutes for each joint.

### **S**tatistical analysis for single-wavelength PAI of arthritis

Aiming at examining the capability of PAI working at 580 nm in differentiating inflammatory arthritic and normal joints, the imaging results from 16 patients and 16 healthy controls were compared. Two parameters both correlated with the increased hemoglobin content (i.e., hyperemia) per unit area in arthritic joints were quantified, including 1) the density of the pseudo-color pixels, and 2) the averaged intensity of the pseudo-color pixels within the joint area. Then, the outcomes from the arthritic and the normal joints were analyzed using the built-in student t-test function in MATLAB (R2010a, Mathworks, Natick, MA). For each parameter (either the density or the average intensity of the pseudo-color pixels), the null hypothesis of the t-test was that the active synovitis and the healthy control cases cannot be differentiated by the quantified parameters extracted from the PA-US combined images.

### Double-wavelength PAI of hemoglobin oxygenation

Two sets of PA images taken at 576-nm and 584-nm wavelengths respectively were used to quantify the spatially distributed hemoglobin oxygenation (sO_2_) in each joint. Simultaneously employing two laser systems which pulse alternatively, the time delay between each pair of images (576 nm and 584 nm) was 100 ms. This relatively short delay between the laser pulses at the two wavelengths can contribute to accurate sO_2_ imaging^35^. In this work, using the described protocol for image scan (i.e., with patient’s hand lying on a flat surface and scanned by a handheld probe), we did not notice obvious motion artifacts between each pair of images.

With the gray-scale US image of each joint obtained, the area of synovium with its boundary delineated by bones and tendon was segmented. The segmentation result was confirmed by a board certificated radiologist. Then the pixel-by-pixel hemoglobin sO_2_ values were calculated using the PA images at the two wavelengths, following the classic method described in the literatures^36,37^. The details about synovium segmentation and calculation of sO_2_ images can be found in the Supplementary Information (S3).

### Statistical analysis for double-wavelength PAI of arthritis

The aim of this statistical analysis is to examine whether double-wavelength PAI can identify the decreased hemoglobin oxygenation as a measurement of hypoxia in arthritic joints compared to normal joints. With the sO_2_ image obtained for each joint, an average sO_2_ value is calculated by performing an averaging over all the pixels in the segmented synovium. The average sO_2_ values from the 10 patient and the 10 health controls were analyzed using the same t-test function in MATLAB, with the null hypothesis that the arthritis patients and the normal subjects cannot be differentiated by using the average hemoglobin sO_2_ in the joints quantified by the double-wavelength PAI.

### Data availability

The data that support the finding of this study are available from the corresponding authors on request.

## Results

In the example result from an arthritic joint, as shown in Fig. 2, strong structural correlations can be found between the US (Fig. 2A) and PA (Fig. 2B) images, as marked by the arrows in the images. Since PA and US images were acquired at the same time by using the same linear probe along the same image plane, they are naturally co-registered, which benefits the interpretation of the imaging findings. In the PA image, besides strong signals from the skin and the phalanges, some strong signals in the area next to the phalanges can be noticed. These signals, which are expected from hyperemia, can be visualized better in the PA-US combined image in Fig. 2C. Taking advantages of the good sensitivity of PAI to blood and the excellent performance of US imaging in describing the morphological tissue structures in the joint, the functional information of hyperemia and its relative position in the joint can be visualized in this combined image. The active inflammation reflected by the PA image was confirmed by the finding from the Doppler US image as shown in Fig. 2D. Due to the incapability to revisit exactly the same imaging plane examined by the US Doppler imaging, there is mismatch between Figs 2C,D. For arthritic MCP joints, most hyperemia exists in the synovium in the form of a sheet of blood or a blob of blood, as illustrated in detail in Fig. S7 in the Supplementary Information. Figure 2C shows a typical case of a sheet of blood. The PA-US combined images of more arthritis MCP joints, including both forms of hyperemia are shown in Fig. S8 in the Supplementary Information.

**Figure 3:**
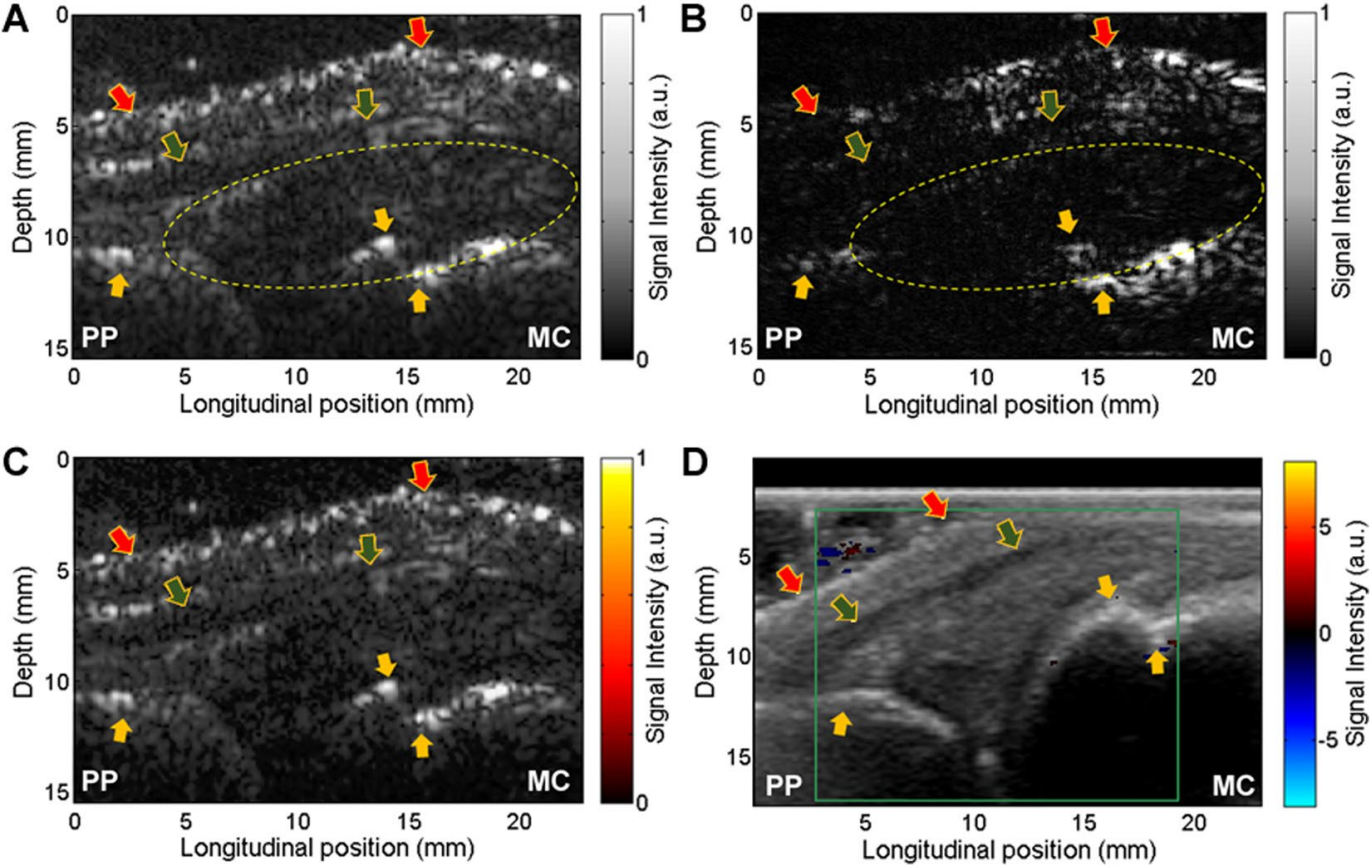
PA and US dual imaging of a right hand 2^nd^ MCP joint of a healthy control subject. PP: proximal phalanges. MC: metacarpals. (**A**) Gray scale B-mode US image. (**B**) Gray scale PA image of the same imaging plane. (**C**) Pseudo-color PA image superimposed on the gray scale US image, processed in the same way as Fig. 2C. (**D**) US Doppler image acquired by a commercial US unit. Yellow arrow: bone; Green arrow: tendon; Red arrow: skin.

Figure 3A,B are the example PA and US images of a healthy joint. Following the same post-processing procedure as that used in Fig. 2, a PA-US combined image was formulated, as shown Fig. 3C. Unlike the result from the arthritis joint, no prominently enhanced hemoglobin signal can be identified in the synovium of the normal joint. This finding was also confirmed by the US Doppler image (Fig. 3D) which does not show any enhanced blood flow in the joint space. The PA-US combined images of healthier joints are shown in Fig. S9 in the Supplementary Information.

To evaluate the sensitivity and specificity of the single-wavelength PA imaging in detecting the hyperemia in the inflammatory arthritic joints, experiments have been conducted on 16 patients with active arthritic and 16 healthy controls. The detailed results are shown in the Supplementary Information (S4). In the combined image from each arthritic joint, we can notice enhanced PA signals (i.e., color pixels) in the joint space. Active synovitis in each joint was confirmed by Doppler US. In comparison, PA-US combined images acquired from the 16 normal joints do not show enhanced PA signals in the joint space. The non-active synovium of each healthy control was also confirmed by Doppler US.

**Figure 4:**
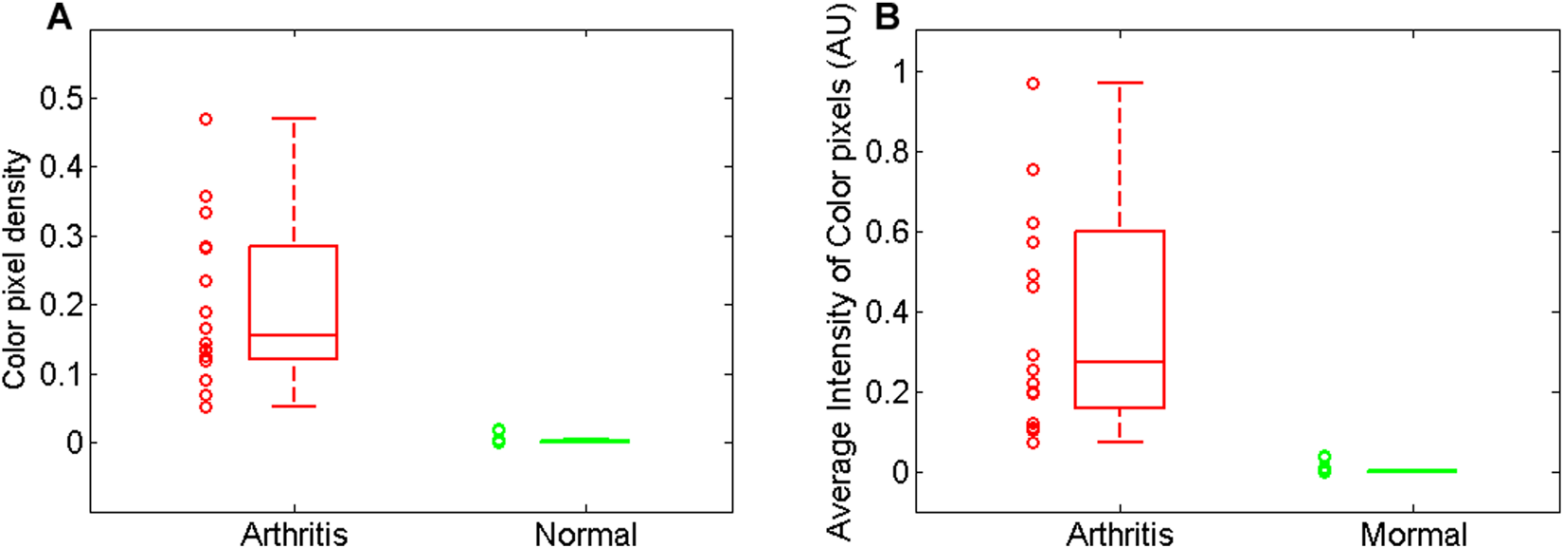
Statistical studies comparing single-wavelength PA imaging results from the 16 inflammatory MCP joints (Arthritis) to those from the 16 healthy MCP joints (Normal). (**A**) The quantified density of the pseudo-color pixels in the joint area. (**B**) The averaged intensity of the pseudo-color pixels in the joint area.

With the PA-US combined images from the 16 arthritis and the 16 normal joints, we statistically studied the two parameters, i.e., the density of the pseudo-color pixels and the averaged intensity of the pseudo-color pixels in the joint area, both quantitatively reflecting hyperemia. For the arthritis group, the average and the standard deviation for the density of the pseudo-color pixels are 0.199 ± 0.117; for the normal group, the average and the standard deviation for the density of the pseudo-color pixels are 0.003 ± 0.006. The box plot showing the densities of the pseudo-color pixels for the two groups is in Fig. 4A. The probability that the two groups cannot be differentiated was assessed using a t-test, and a p < 0.001 was obtained. With the averaged intensities of the pseudo-color pixels from the 16 joints in each group quantified, an average and a standard deviation were calculated. The results for the arthritis group and the normal groups were 0.444 ± 0.420 and 0.006 ± 0.013, respectively. The box plot showing the averaged intensities of the pseudo-color pixels for the two groups is in Fig. 4B. The probability that the two groups cannot be differentiated was assessed using a t-test, and a p < 0.001 was obtained. We also evaluated the post-hoc power using the above results and the sample size used (n = 16). The calculated effect sizes were large (Cohen’s d = 2.37 for density of the pseudo-color pixels, and 1.47 for averaged intensities of the pseudo-color pixels), and the achieved power was greater than 0.98 on each outcome.

**Figure 5:**
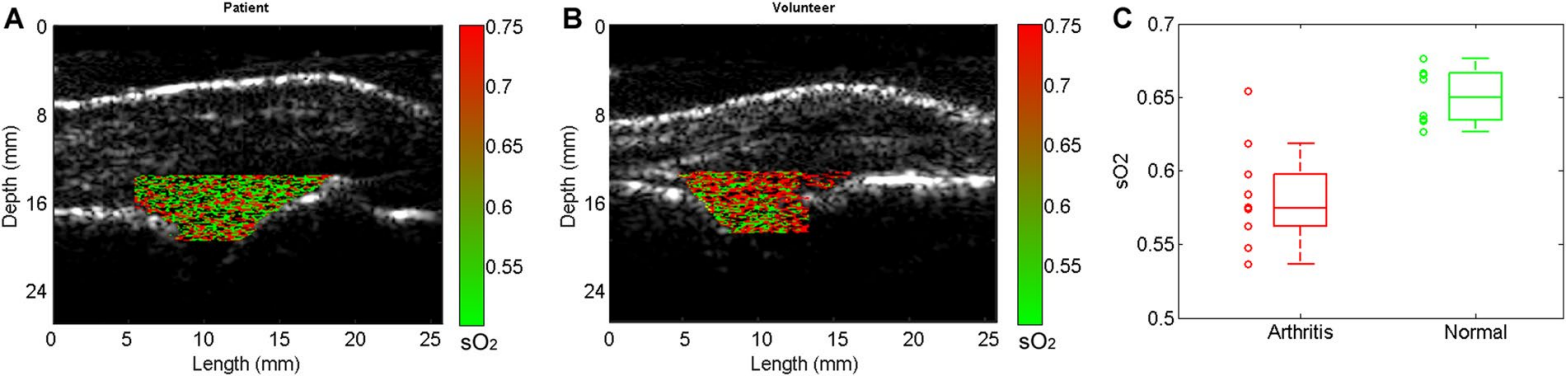
PA hemoglobin oxygenation (sO_2_) image superimposed on the gray-scale US image of human MCP joint. (**A**) Imaging result from an unequivocal inflammatory MCP joint. (**B**) Imaging result from a healthy MCP joint. (**C**) Averaged sO_2_ levels in the joint space: 10 unequivocal inflammatory joints (Arthritis) vs. 10 healthy joints (Normal).

By conducting double-wavelength PA imaging of arthritic joints and normal joints, decreased hemoglobin oxygenation (i.e., hypoxia) in synovium as another physiological biomarkers of synovitis was studied. Figure 5(A),(B) shows example PA hemoglobin oxygenation (sO_2_) images superimposed on the gray-scale US images of MCP joints. Difference in sO_2_ levels between the arthritis and the normal joints can be recognized. With the imaging results from the 10 arthritis joints and the 10 normal joints, a t-test was conducted to examine whether the quantified hemoglobin sO_2_ extracted from the double-wavelength PA images can differentiate the two groups, and a p < 0.001 was achieved. The averaged hemoglobin sO_2_ levels are 0.582 ± 0.034 and 0.651 ± 0.020 for the arthritic joints and the normal joints, respectively. The box plot showing the hemoglobin sO_2_ for the two groups of joints is in Fig. 5C.

## Discussion

The findings from the single-wavelength and the double-wavelength PAI on human subjects in this study agree with the fact of increased vasculature, intensified tissue metabolism and consequently hypoxia in the arthritic finger joints. When working with a single laser wavelength, PAI has demonstrated prominent sensitivity to the increased vascularity (i.e., hyperemia) in the arthritic finger joints. With 16 subjects in each group, statistical significance has been achieved in differentiating patients and healthy control subjects. When working with two laser wavelengths, PAI has demonstrated unique capability of quantifying decreased hemoglobin oxygenation (i.e., hypoxia) in local joint space. With 10 subjects in each group, double-wavelength PAI has shown statistical significance in differentiating patients and healthy control subjects.

The result from this study on human subjects suggests that PAI, as a complement to musculoskeletal US, could enable the study of additional physiology biomarkers of inflammatory arthritis *in vivo*, in a non-ionizing and non-invasive manner. Although hypoxia is an important physiological biomarker of synovitis reflecting the increased metabolic demand and the relatively inadequate oxygen delivery of the inflammatory synovial tissue, quantitatively evaluating hypoxia *in vivo* is still challenging and is not feasible by traditional US imaging. In addition, unlike Doppler US which detects the vasculature in synovial tissue based on the measurement of blood flow toward or away from the probe, PAI of vessels is instead targeting the optical absorption of hemoglobin. Vessels in any size, with any flow velocity, and along any orientation can be detected by PAI, as long as they contain hemoglobin which produces PA signals when illuminated by light. Therefore, PAI of vasculature could be less operator-dependent, and can potentially be more quantifiable.

PA modality combined with established US imaging holds potential to resolve the challenges in rheumatology clinic in diagnosis and treatment assessment of inflammatory arthritis. Based on the highly sensitive optical contrast, PAI may enable earlier diagnosis and earlier initiation of treatment leading to better remission rates. In addition, PAI may contribute to the assessment of treatment response, e.g., early identification of non-responders which can facilitate immediate course correction, thereby promoting better patient care and reducing unnecessary morbidity and cost.

The prototype, yet highly translational PA-US dual-modality system developed and tested in this study could accommodate routine arthritis screening in clinics. Structured within the framework of conventional US scanning procedures, the handheld probe integrates all imaging components including the fiber optics and the US transducer array. This integrated design minimizes the need for optical and acoustic alignment and thereby maximizes scanning flexibility. The acoustic reflecting imaging geometry allows direct light illumination on the imaged tissue volume, improving optical energy efficiency and reducing potential risk of burning the skin. However, a drawback of such design is that the acoustic mismatch between the glass and the US gel could lead to phase distortion and reduce the image quality in beamforming based reconstruction. Better acoustic reflecting and optically transparent materials, such as those used in the literature^38^, will be investigated for more desirable image quality. Through the theoretical analysis and the experimental validation, the optical wavelengths were optimized for PAI of finger joints. A balance between the imaging depth and the imaging sensitivity was achieved. Although light penetration at these optical wavelengths is limited, the imaging depth is sufficient for covering the small joints of human hands. In the future, when larger joints (e.g., knees and ankles) are the targets, wavelengths in the optical spectrum of 650–950 nm enabling better light penetration could be better options.

Our prototype PA-US system was not capable of Doppler US imaging. A commercial Doppler US unit was thereby employed as the gold standard for identifying arthritis patients and healthy control subjects. The image procedure involving two US scanners determined the incapability to visit exactly the same plane by US Doppler and PA-US dual imaging. As another part of future study, US Doppler function will be integrated into our PA-US system for more accurate comparison between the modalities.

## Electronic supplementary material

Supplementary Information
