# Supplementary material for: A Functional Study of Human Inflammatory Arthritis Using Photoacoustic Imaging
Source: Sci Rep. 2017 Nov 3;7:15026. doi: 10.1038/s41598-017-15147-5 (PMC5670248; doi:10.1038/s41598-017-15147-5)
Supplement: Supplementary file 1 — Supplementary Information [file 41598_2017_15147_MOESM1_ESM.pdf]

SUPPLEMENTARY INFORMATION

A Functional Study of Human Inflammatory Arthritis Using Photoacoustic Imaging

Janggun Jo[1,&]

Guan Xu[2,&]

Meng Cao[3]

April Marquardt[4]

Sheeja Francis[4]

Gandikota Girish[2,*]

Xueding Wang[1,2,*]

[1]Department of Biomedical Engineering, University of Michigan, Ann Arbor, Michigan 48109

[2]Department of Radiology, University of Michigan Medical School, Ann Arbor, Michigan 48109

[3]Department of Electronic Science and Engineering, Nanjing University, Nanjing, China 210093

[4]Division of Rheumatology, Department of Internal Medicine, University of Michigan School of Medicine, Ann Arbor, Michigan 48109

[&] These authors contributed equally to the work.

*** Corresponding authors: Gandikota Girish (email: ggirish@umich.edu) and Xueding Wang (email: xdwang@umich.edu). University of Michigan, 2125 Gerstacker Building, 2200 Bonisteel Blvd, Ann Arbor, MI 48109, USA. Fax: 734-936-1905*

S1. Selection of wavelengths for photoacoustic imaging (PAI) of arthritis

Before starting this study on human subjects, the optical wavelength for the specific scenario of PAI of human finger joint was optimized based on theoretical analysis and the experiment on a cadaver hand. The purpose of the experiment is to find the wavelengths that can enable sufficient penetration in the finger join while providing good photoacoustic (PA) imaging contrast between the blood in joint space and the bone within the finger. The optical absorption of both oxygenated and deoxygenated hemoglobin have a predominantly decreasing trend in the visible to near-infrared (NIR) spectral range [1]. The optical illumination at shorter wavelength ensures higher signal-to-noise ratio (SNR) in detecting blood whereas the optical illumination at longer wavelength facilitates deeper penetration in optically scattering soft tissues.

Fig. S1(a) shows the optical absorption spectra of bone [2,3], oxygenated hemoglobin (Oxy-Hb), and deoxygenated hemoglobin (Deoxy-Hb) [1], all having overall descending trends in the spectral range of 500-1000 nm. The optical absorption contrasts of oxygenated and deoxygenated hemoglobin over bone, as shown by the dashed curves in Fig. S1a, reach maxima within the range of 560-590 nm. We therefore limited the search for optimal wavelength(s) for joint imaging in this range.

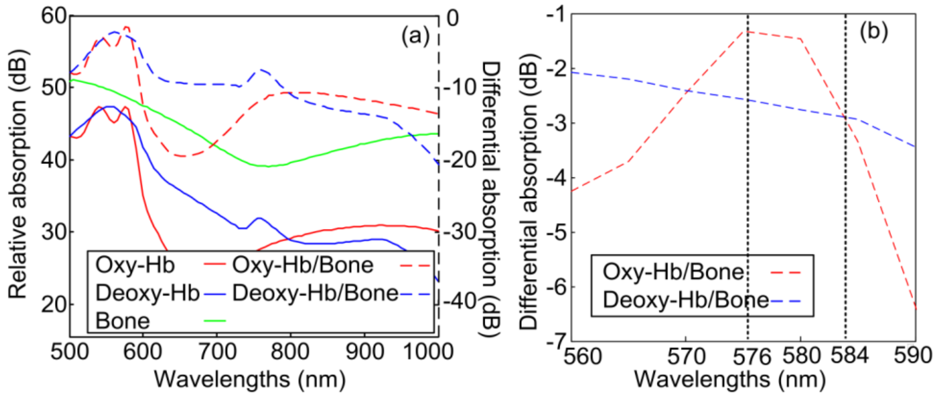

Figure S1. (a) Relative optical absorption spectra of bone, oxygenated hemoglobin (Oxy-Hb), and deoxygenated hemoglobin (Deoxy-Hb). The contrasts of the two forms of hemoglobin over bone as functions of optical wavelength are also shown. (b) Magnified spectra showing the wavelength dependent optical absorption contrasts of oxygenated and deoxygenated hemoglobin over bone in the range of 560-590 nm.

A cadaver hand was used to further identify the optimal wavelength for PAI of arthritis in human peripheral joints. During the experiment, 0.1 ml fresh blood was injected into the metacarpophalangeal (MCP) joints in the cadaver hand under the guidance of ultrasound (US) imaging. All the 5 MCP joints were used. The PA images of each joint were acquired at 570 nm, 580 nm, and 590 nm. Fig. S2 shows the example imaging result from one of the MCP joints. The bone areas were picked up manually based on the parallel US image of the joint, as shown in Fig. S2(a). The needle track during blood injection was observed under the US. The bright pixels around the needle tracks in PA images representing the blood injected were selected, as marked by the red dashed circles in the images. To quantify the enhancement in PA intensity at each wavelength brought by the injected blood, the ratio between the average pixel intensity within the blood injection areas (i.e., the areas marked by the red dashed circles) and the average pixel intensity in a randomly selected area out of the blood injection area, i.e., the area marked by the blue dashed square in Fig. S2(b)-(d), was calculated. The quantified ratios at the three wavelengths (570 nm, 580 nm, and 590 nm) are 2.5, 3.9, and 1.9, respectively. The image depths at all the three wavelengths are sufficient for visualizing the injected blood in the MCP joints. To quantify the contrast of the injected blood over the bone in each PA image, the ratio between the average pixel intensity within the blood injection areas (i.e., the areas marked by the red dashed circles) and the average pixel intensity within the bone areas (i.e., the areas marked by the yellow dashed circle) was calculated. As each wavelength, the ratios from the 5 MCP joints were averaged and the standard deviation was computed. The results for the three wavelengths were compared in Fig. S3. The image contrast between the injected blood over the bone reached a peak at 580-nm wavelength which was then chosen for the study on human subjects involving single-wavelength PAI.

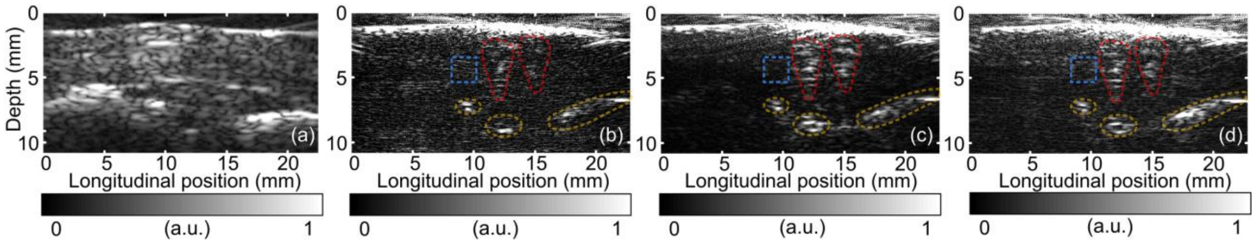

Figure S2. Representative US and PA images of a cadaver finger joint. (a) Gray-scale US image of the joint facilitating the recognition of bones (marked by the yellow dashed circles). (b)-(d) PA images of the cadaver finger joint acquired at 570 nm, 580 nm, and 590 nm, respectively. Guided by US imaging, 0.1 ml fresh blood was injected in the joint region just before the imaging. The areas for blood injection are marked by the red dashed circles. The blue dashed square in (b)-(d) indicates an area out of the blood injection areas to be considered as a background.

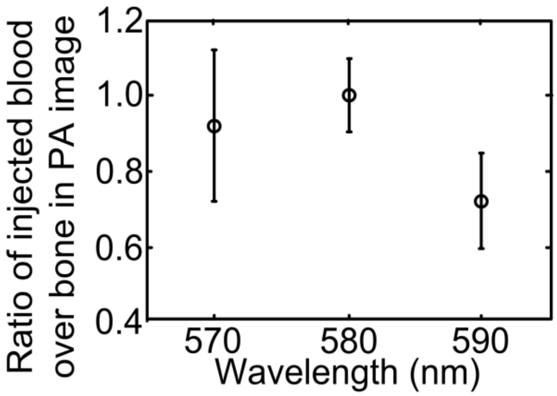

Figure S3. Statistical analysis in search for the optimal illumination wavelength for PAI of arthritis in human finger joint. The ratios between the average pixel intensity within the blood injection areas and the average pixel intensity within the bone areas were quantified for PA images acquired from the five MCP joints of a cadaver hand. The average and the standard deviation at each of the three wavelengths (i.e., 570 nm, 580 nm, and 590 nm) were calculated for comparison.

For double-wavelength PAI aiming at evaluating hypoxia as the second physiological biomarker of arthritis, the wavelengths of 584 nm and 576 nm, both close to the wavelength of 580 nm determined in the experiment on the cadaver hand, were selected. At 584 nm, the optical absorption coefficients of the oxygenated and deoxygenated hemoglobin are equal (i.e., an isosbestic point). At 576 nm, the contrast between oxygenated hemoglobin over bone has a local peak, as shown in Fig. S1b. PAI of a target joint at these two wavelengths can facilitate the separation of the two forms of hemoglobin as well as the quantification of hemoglobin oxygenation in the joint. We intentionally selected the two optical

wavelengths that are close, because largely separated two wavelengths can lead to significantly different optical attenuation in tissue and, therefore, difference in optical penetration. This difference, if not compensated, may affect the accuracy in quantitative evaluation of hemoglobin sO_2 by using multi-wavelength PAI. In the future, to achieve more accurate sO_2 imaging in the joint, the wavelength-dependent light attenuation can be compensated via the established methods [4-6].

S2. Selection of cutoff threshold to isolate the vasculature in the joint

As mentioned in the manuscript, before a single-wavelength PA image is co-registered with an US image acquired from the same joint, a cutoff at 0.4 is performed to isolate the vasculature and remove the background and the noise in the normalized PA image. Such filtering technique is similar to the wall filter that is used in Doppler US for removing the artifacts due to the motions in background tissue as well as system noise [7]. Similarly, the cutoff threshold adopted in PAI removes the pixels representing background tissue and system noise, retaining only those correlated with the increased hemoglobin content, i.e., hyperemia.

After filtering by performing the cutoff, the number of remaining color pixels in the PA-US combined image, as an example shown in Fig. 2c, quantifies the total area of vascularity within the imaged plane. The optimal cutoff threshold is thereby the value at which the PA images from patients and normal controls have the maximum difference in terms of the vascularity in joint space as reflected by the PA images. Using the data acquired from 16 arthritic joints and 16 normal joints, the cutoff value was searched between 0.2 and 0.7, with a constant step size of 0.1. At each step, the probability (p-value) that the two conditions (arthritis vs. normal) cannot be differentiated based on the density of the pseudo-color pixels in the PA-US combined images was calculated, as shown in Fig. S4. The cutoff value at 0.4 produced the least p-value, i.e., the best differentiation of arthritis joints and normal joints.

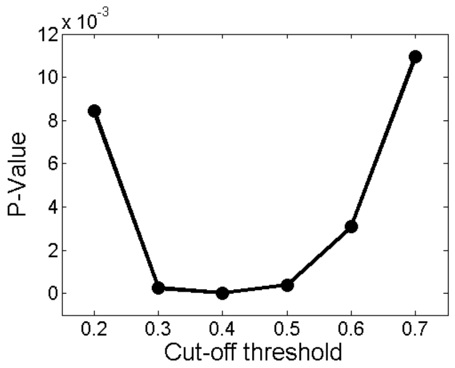

Figure S4. Optimization of the cutoff threshold for extracting the vascular pixels in the PA image from each joint. To find out the best cutoff threshold, the p-value for differentiating the 16 arthritic and the 16 normal joints based on the density of the pseudo-color pixels in the PA-US combined image was calculated for each cutoff value from 0.2 to 0.7. The least p-value appears when the cutoff is set at 0.4.

S3. Procedure for calculating hemoglobin oxygenation (sO_2) in synovium

Before calculating the hemoglobin sO_2 image, the synovium segmentation of each joint was conducted based on B-scan US images acquired at the same time of dual-wavelength PAI. The gray scale US image was first denoised using Gaussian filter, as shown in Fig. S5. The denoised image would help in recognizing the boundaries of bones and tendon in the joint. Then the area of synovium, i.e., the region of interest (ROI) to be studied, with its boundary delineated by the metacarpal head, the phalanx and the tendon was segmented. The segmentation results were confirmed by a board certificated radiologist.

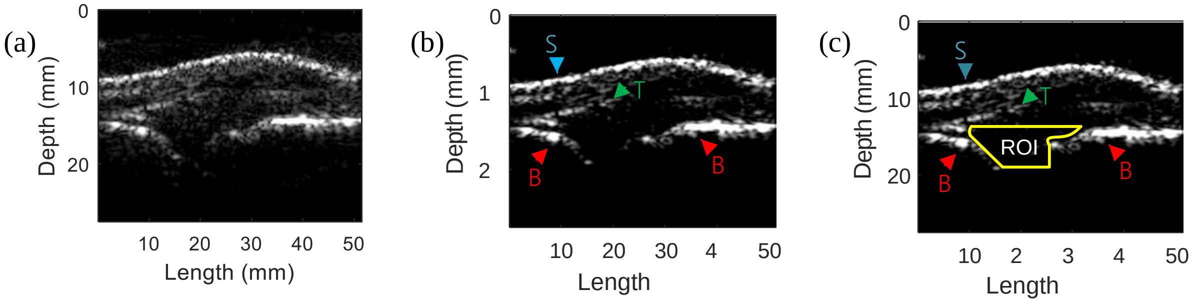

Figure S5. (a) Gray-scale US image of a human finger joint. (b) The denoised US image with clearer boundaries. S: skin; T: tendon; B: bone. (c) The synovium segmented from the bionarized US image of the joint (i.e., the ROI for calculation of sO_2).

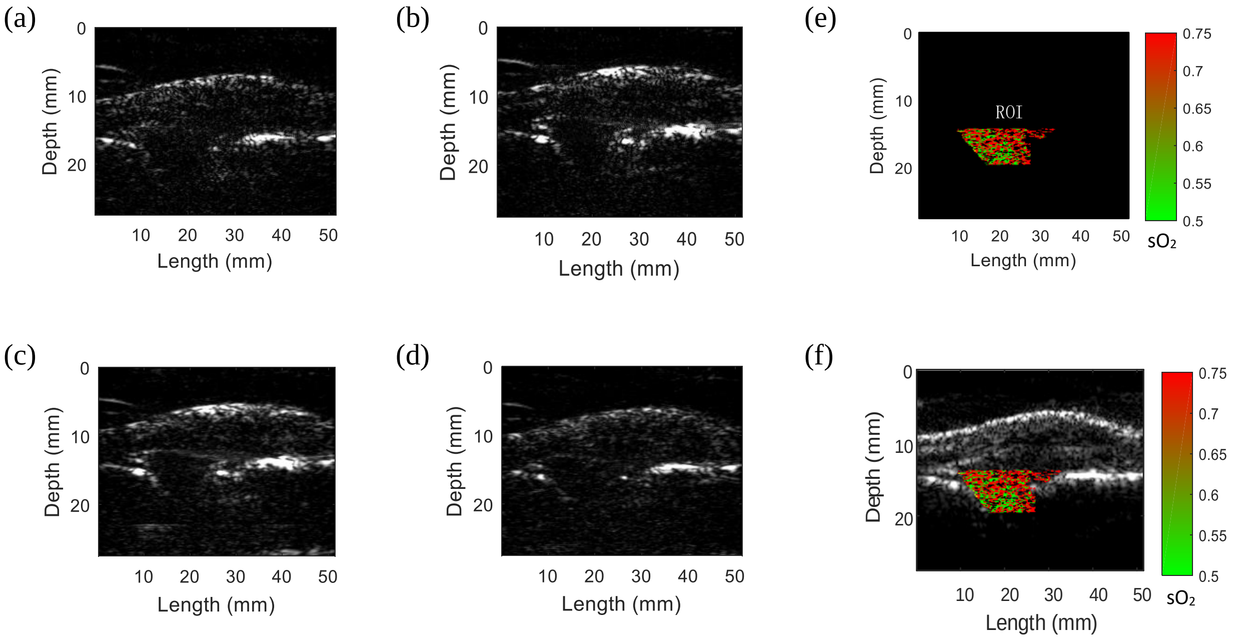

Figure S6. (a) and (b) Gray-scale PA images from a finger joint acquired at 576-nm and 584-nm wavelengths, respectively. Using Gaussian filter, the two images were denoised, as shown in (c) and (d), before being used for calculation of hemoglobin sO_2. (e) The pixel-by-pixel hemoglobin sO_2 image in the ROI calculated from the PA images at the two wavelengths. (f) The pseodu-color PA sO_2 image super-imposed on the gray-scale US image.

The PA images at the two wavelengths (i.e., 576 nm and 584 nm) acquired from the same joint are shown in Fig. S6. Before calculation of sO_2, each image was denoised by using Gaussian filter. With the two PA images, the pixel-by-pixel blood sO_2 in the ROI was then calculated, by using the following equations,

$$\mathrm{P}_{(576)} = \mathrm{K}(\alpha_{HbO_2\,(576)} \cdot C_{HbO_2} + \alpha_{\,Hb(576)} \cdot C_{Hb}) \tag{1}$$

$$\mathrm{P}_{(584)} = \mathrm{K}(\alpha_{HbO_2\,(584)} \cdot C_{HbO_2} + \alpha_{\,Hb(584)} \cdot C_{Hb}) \tag{2}$$

where $P_{(576)}$ and $P_{(584)}$ are the corresponding photoacoustic pixel intensities measured at 576 nm and 584 nm, relatively, K is a system-related constant, α_{HbO_2} and α_{Hb} are the molar extinction coefficients (cm^{-1} M^{-1}) of oxygenated hemoglobin (HbO_2) and deoxygenated hemoglobin (Hb), respectively, C_{HbO_2} and C_{Hb} are the concentrations of the two forms of hemoglobin, respectively. The hemoglobin sO_2 is defined as

$$sO_2 = \frac{C_{HbO_2}}{C_{HbO_2}+C_{Hb}} \quad (3).$$

From Eq. (1) and (2), and the pixel-by-pixel hemoglobin sO_2 can be calculated by

$$sO_2 = \frac{P_{(576)} \cdot \alpha_{Hb(584)} - P_{(584)} \cdot \alpha_{Hb(584)}}{P_{(576)}\left(\alpha_{Hb(584)} - \alpha_{HbO_2(584)}\right) + P_{(584)}\left(\alpha_{Hb(584)} - \alpha_{HbO_2(584)}\right)} \quad (4).$$

For each joint, 10 pairs of 576-nm and 584-nm PA images were acquired. Then, the 10 hemoglobin sO_2 images calculated for the joint were averaged to reduce noise. The obtained PA functional image reflecting the pixel-by-pixel hemoglobin sO_2 in the synovium was presented in pseudo-color and then super-imposed on the gray-scale US image, as shown in Fig. S6f. To make this method of double-wavelength PAI of hemoglobin sO_2 work, one assumption is that the spatial distributions of light fluence in the sample at the two wavelengths are similar (or can be measured for later compensation by dividing the image intensities by the light fluence). This assumption can be met in this study considering that the two wavelengths (i.e., 576 nm and 584 nm) selected for double-wavelength PAI of human finger joint are very close, ensuring similar attenuation and spatial distributions of the light fluence at the two wavelengths.

S4. PA-US combined images of human finger joints from 16 patients and 16 healthy controls

Fig. S7 shows a sketch of an inflammed human finger joint with the hyperemia in synovium marked. Most hyperemia exists in the synovium in the form of a sheet of blood or a blob of blood. Fig. S8 shows the PA-US combined images of 16 arthritic joints. All PA images were acquired at a single-wavelength of 580 nm. The pseudo-color PA image superimposed on the gray-scale US image was processed following

the same post-processing procedure as that used in Fig. 2. images were obtained with single –wavelength PAI. And, the 16 healthy joints' images were also collected. In comparison with the sketches in Fig. S7, the form of hyperemia was described in Fig. S8 caption.

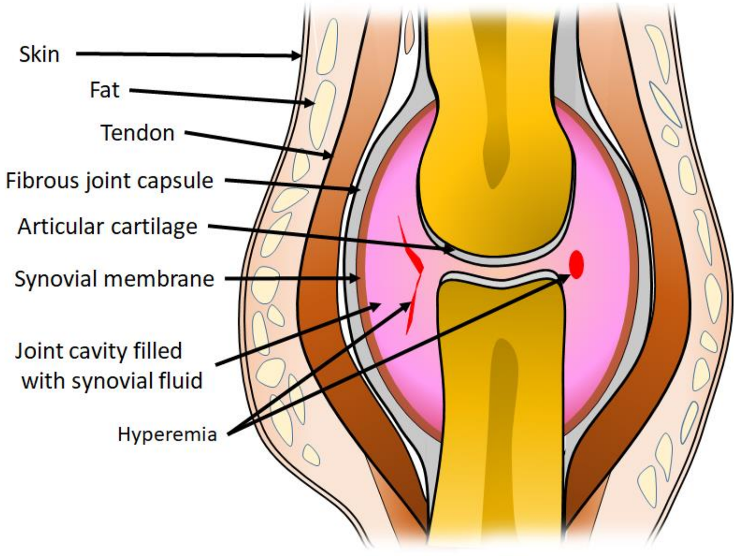

Figure S7. Sketch of an inflammed human finger joint with hyperemia in synovium.

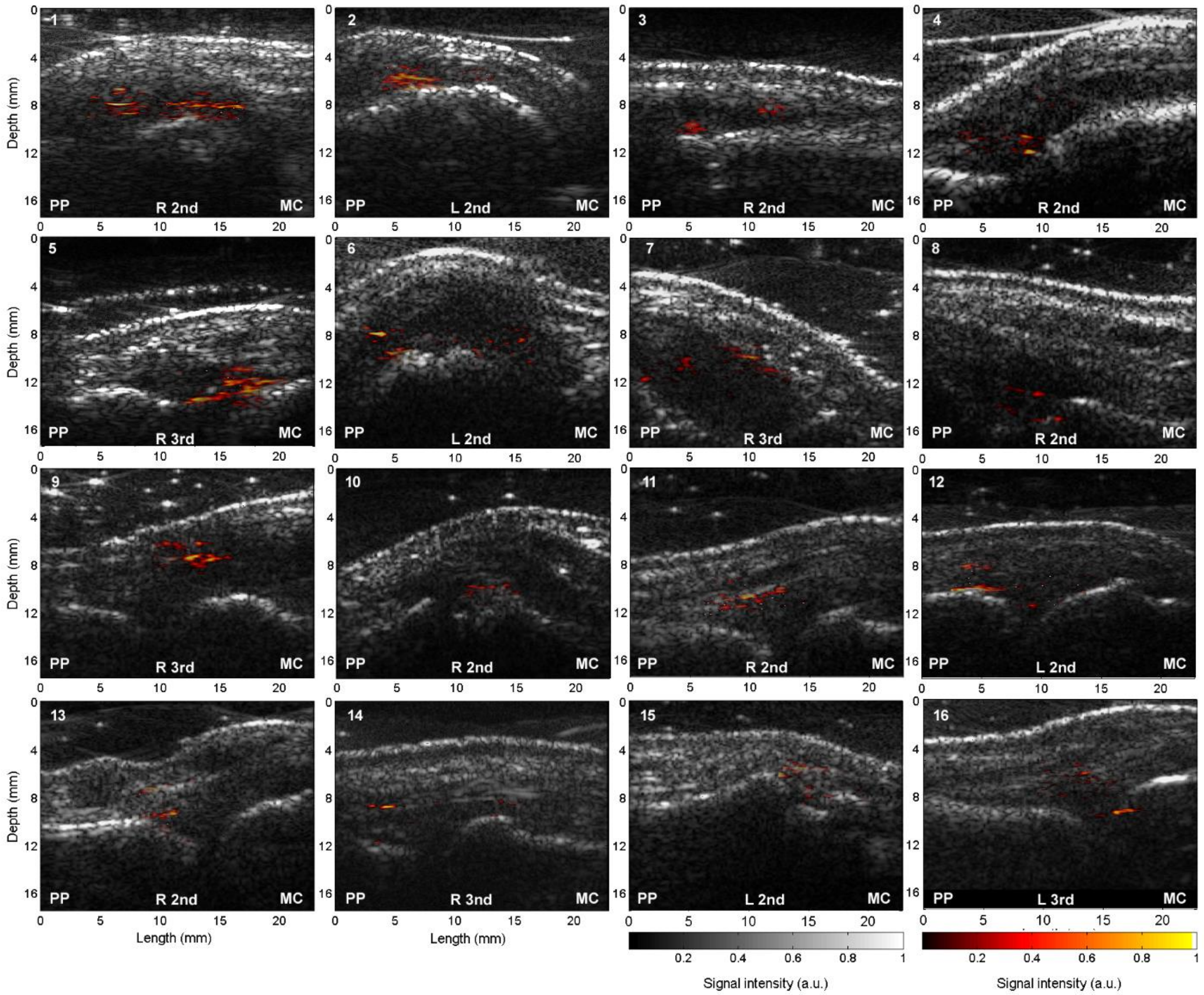

Figure S8. PA-US combined images of 16 MCP joints affected by inflammatory arthritis. PP: proximal phalanges. MC: metacarpals. R: right hand. L: left hand. Cases 1, 2, 8, 11, 12 and 13 are with hyperemia in sheet form, cases 3, 4, 6, 15 and 16 are with hyperemia in blob form, and cases 5, 7, 9, 10, 14 have both forms of hyperemia existed.

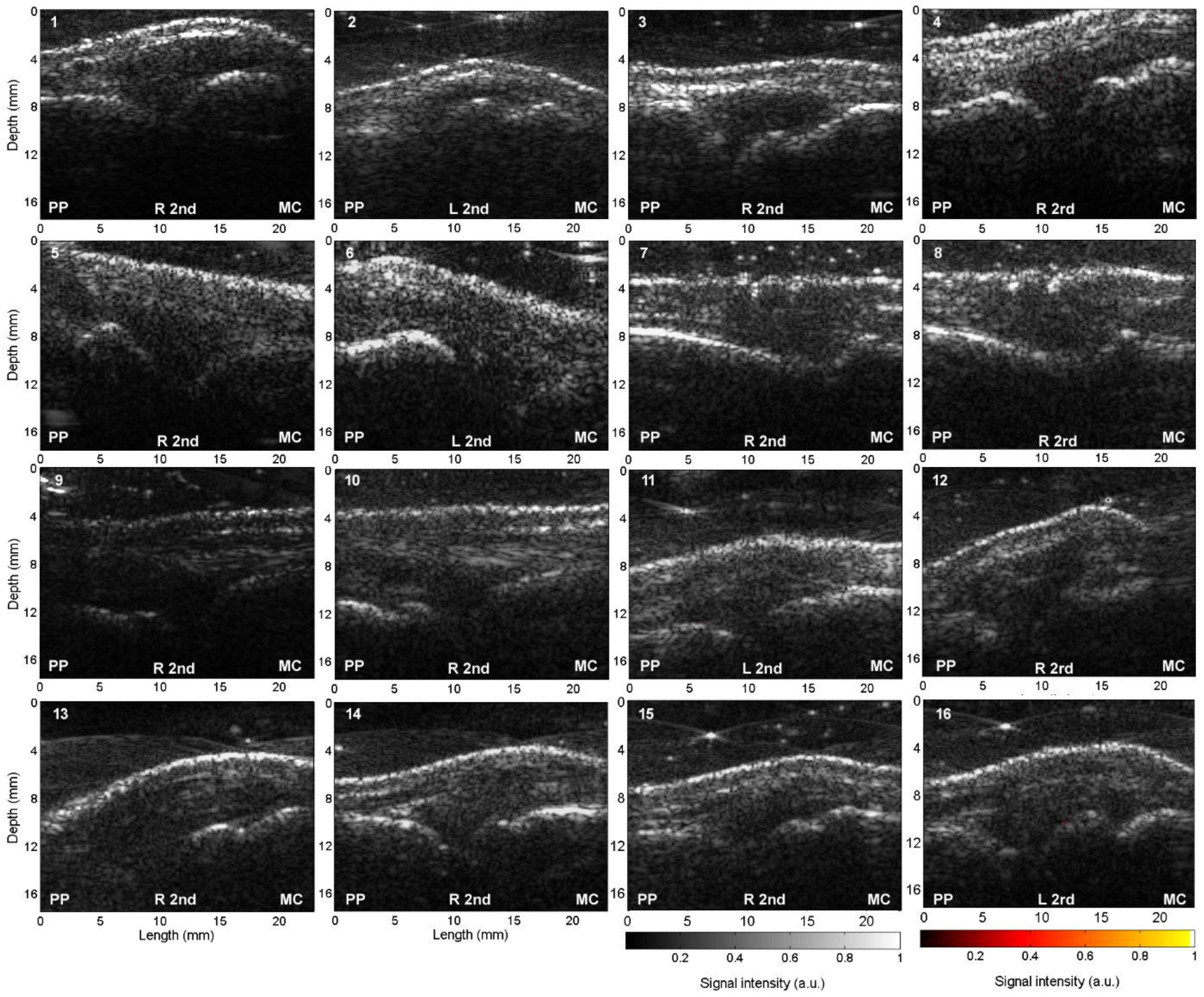

Figure S9. PA-US combined images of 16 MCP joints from normal volunteers. PP: proximal phalanges. MC: metacarpals. R: right hand. L: left hand.

References:

1 Jacques, S. L. Optical properties of biological tissues: a review. *Physics in Medicine and Biology* **58**, R37 (2013).

2 Bashkatov, A. N., Genina, E. A., Kochubey, V. I. & Tuchin, V. V. 616310-616310-616311.

3 Feng, T. *et al.* Bone assessment via thermal photo-acoustic measurements. *Optics letters* **40**, 1721-1724, doi:10.1364/ol.40.001721 (2015).

4 Dean-Ben, X. L., Buehler, A., Ntziachristos, V. & Razansky, D. Accurate model-based reconstruction algorithm for three-dimensional optoacoustic tomography. *IEEE Trans Med Imaging* **31**, 1922-1928, doi:10.1109/TMI.2012.2208471 (2012).

5 Brochu, F. M. *et al.* Towards Quantitative Evaluation of Tissue Absorption Coefficients Using Light Fluence Correction in Optoacoustic Tomography. *Ieee T Med Imaging* **36**, 322-331, doi:10.1109/Tmi.2016.2607199 (2017).
6 Tzoumas, S. *et al.* Eigenspectra optoacoustic tomography achieves quantitative blood oxygenation imaging deep in tissues. *Nat Commun* **7**, 12121, doi:10.1038/ncomms12121 (2016).
7 Bude, R. O. & Rubin, J. M. Power Doppler sonography. *Radiology* **200**, 21-23 (1996).
